# Genetic structure and phylogenetic analysis of grapevine leafroll-associated virus-1 (*Ampelovirus univitis*) in different grape-producing regions of Iran

**DOI:** 10.1128/spectrum.01528-25

**Published:** 2025-10-27

**Authors:** Nesa Razavi, Davoud Koolivand, Masoud Naderpour, Milad Yousefi

**Affiliations:** 1 Department of Plant Protection, Faculty of Agriculture, University of Zanjan 185134, Zanjan, Iran; 2 Seed and Plant Certification and Registration Research Institute (SPCRI), Agricultural Research, Education and Extension Organization (AREEO), Karaj, Iran; USDA-ARS-NPRL, Dawson, Georgia, USA

**Keywords:** GLRaV-1, phylogenetic analysis, genetic structure, grapevine viruses, Iran, population diversity, virus evolution

## Abstract

*Grapevine leafroll-associated virus 1* (GLRaV-1) is a major viral pathogen within the genus *Ampelovirus* and a key contributor to grapevine leafroll disease, posing significant threats to viticulture worldwide. Despite its importance, the genetic structure and diversity of GLRaV-1 in Iran have remained largely unexplored. In this study, a total of 325 symptomatic grapevine samples were collected from 5 major grape-producing provinces of Iran and screened using immunoassay and reverse transcription-PCR (RT-PCR) for GLRaV-1. Nine positive samples were subjected to sequencing of the coat protein (CP) gene. Phylogenetic analyses of CP gene sequences and publicly available full-genome sequences, performed using MEGA 11, revealed three distinct phylogenetic clades among Iranian isolates. Recombinant sequences were identified and excluded to avoid phylogenetic bias. Population genetic analyses indicated high haplotype diversity and strong purifying selection across the genome. Significant population structure and restricted gene flow were observed, consistent with geographic isolation of viral populations. Molecular clock analysis suggested recent divergence of GLRaV-1 lineages within Iran. These findings provide new insights into the evolutionary dynamics of GLRaV-1 and its population structure in Iranian vineyards, with implications for virus spread, surveillance, and disease management.

**IMPORTANCE:**

Grapevine leafroll-associated virus 1 (GLRaV-1) is a widespread and economically important virus affecting grapevines. This study is the first to investigate the genetic diversity and population structure of GLRaV-1 across major grape-growing regions of Iran. We discovered a high level of genetic variation and geographically structured virus populations, which may reflect localized transmission and limited movement of infected material. Understanding this diversity is crucial for improving diagnostic strategies and managing the spread of grapevine leafroll disease. Our findings support the need for region-specific disease control efforts and contribute to the global understanding of *Ampelovirus* evolution.

## INTRODUCTION

Grapevine (*Vitis vinifera* L.), a member of the family *Vitaceae* and genus *Vitis*, is among the oldest and most economically significant fruit crops worldwide. Its cultivation dates back to ancient times, with origins tracing to the Caucasus region. Grapes play a crucial role in global agriculture, contributing substantially to the food, wine, and juice industries (1). According to the Food and Agriculture Organization (FAO), global grape production reached approximately 78 million tons in 2021. In Iran, vineyards cover an area of 255,160 hectares, with an annual production of 2,775,411 tons.

Despite its economic importance, grapevine production faces numerous challenges, particularly from biotic stressors such as fungi, bacteria, nematodes, and viruses. Among these, viral infections represent a major threat to vineyard health, affecting vine vigor, reducing yield, and compromising fruit quality. More than 80 viruses have been reported to infect grapevines, with some of the most economically devastating species including *Grapevine fanleaf virus* (GFLV), *Arabis mosaic virus* (ArMV), and the *Grapevine leafroll-associated viruses* (GLRaVs) (2). Of these, grapevine leafroll disease (GLRD) is one of the most destructive viral diseases, first reported in the mid-19th century in California (3). It is estimated that GLRD can cause a 30%–50% reduction in grape yield, significantly impacting fruit color, sugar content, and overall marketability (4, 5).

The causative agents of GLRD, known as *Grapevine leafroll-associated viruses* (GLRaVs), belong to the family *Closteroviridae*. This family includes several genera such as *Ampelovirus, Closterovirus, Crinivirus,* and *Velarivirus* (6). To date, at least 13 GLRaV species have been identified, with ongoing research indicating that this number may continue to increase as advanced molecular detection techniques become more widely implemented (7). Among these, *Grapevine leafroll-associated virus-1* (GLRaV-1) is considered a major pathogen in viticulture, ranking second in economic importance after GLRaV-3 (8).

GLRaV-1 possesses a positive-sense single-stranded RNA genome of approximately 18.7-18.9 kb, encapsidated within flexuous, filamentous virions (9). The viral genome comprises nine open reading frames (ORFs) that encode proteins responsible for replication, movement, encapsidation, and vector transmission (10). The virus spreads predominantly through vegetative propagation, including grafting, and is transmitted by mealybug species such as *Planococcus ficus* and *Pseudococcus longispinus* in a semi-persistent manner (11). These transmission routes facilitate its rapid dissemination within vineyards, leading to significant economic losses in both table grape and wine grape industries.

GLRaV-1 has been reported in major grape-producing countries, including Italy, France, Spain, Greece, Germany, Switzerland, Slovakia, Tunisia, and Turkey (12–17). In Iran, extensive surveys and molecular studies have confirmed its widespread occurrence, particularly in the Fars province, one of the country’s key viticultural regions (2, 18). Phylogenetic analyses have revealed substantial genetic diversity among GLRaV-1 isolates, with multiple lineages circulating globally.

Recent research from the United States, China, Italy, Turkey, and Russia have demonstrated that GLRaV-1 isolates cluster into distinct phylogenetic groups (16). However, studies have not established a clear correlation between genetic diversity and geographic distribution, suggesting that other factors, such as vector specificity and vineyard management practices, may influence viral diversity (4, 8, 19–21). Despite multiple reports on GLRaV-1 worldwide, comprehensive studies on its genetic diversity and population structure in Iran remain limited, underscoring the need for further investigation.

Given the significant impact of GLRaV-1 on grapevine yield and quality, and the lack of phylogenetic data from Iran, this study investigates the genetic diversity of GLRaV-1 isolates from Iranian vineyards. By sequencing key genomic regions and comparing them with global isolates, we aim to clarify the virus’s evolutionary patterns and inform more effective management strategies.

## MATERIALS AND METHODS

### Sampling

During the 2023 growing season, a total of 325 samples were collected from symptomatic grapevines across five Iranian provinces: Kohgiluyeh and Boyer-Ahmad (136 samples), West Azerbaijan (68 samples), Khorasan Razavi (59 samples), Qazvin (34 samples), and Fars (28 samples) (Tables 1 and 2). From each vine, one fully developed compound leaf was sampled from the mid-canopy. Leaf samples were collected from grapevines showing symptoms such as mosaic patterns, leaf rolling, chlorosis, reddening, deformation, stunted growth, and uneven berry size. Samples were transported in cool boxes and stored at 4°C prior to analysis. Each sample was assigned a unique number based on the time and date of collection and immediately frozen at −80°C for subsequent total RNA extraction. Details of the collection locations, including GPS coordinates and number of samples per region, are provided in Table 2.

**TABLE 1:** Properties of GLRaV-1 isolates that have been used for analysis in this study^*a*^^,^^*b*^

Collection Date	Accession Number	Host	Isolates/strains	Country
**2023, 2023, 2023, 2023, 2023, 2023, 2023, 2023, 2023**, 2009, 2018, 2018, 2018, 2018, 2018, 2018,	**PP213141, PP213142, PP213143, PP213144, PP213145, PP213146, PP213147, PP213148, PP182259**, FJ952151, OQ849147, OQ849148, OQ849149, OQ849150, OQ849151, OQ849152	*Vitis vinifera* **,** *Vitis vinifera* **,** *Vitis vinifera* **,** *Vitis vinifera* **,** *Vitis vinifera* **,** *Vitis vinifera* **,** *Vitis vinifera* **,** *Vitis vinifera* **,** *Vitis vinifera* **,***Vitis vinifera*, *Vitis vinifera, Vitis vinifera, Vitis vinifera, Vitis vinifera, Vitis vinifera, Vitis vinifer*a	**KG76, KG74, KG64, KG56, KG31, K78, K27, A24, KG75**, IR-S7, KaA42, KaA62, KaA82, KaA92, KaA52, KaA72,	Iran
2013, 2013, 2013, 2013, 2013,	KP067340, KP067341, KP067346, KP067352, KP067369KP067369	*Vitis vinifera, Vitis vinifera, Vitis vinifera, Vitis vinifera, Vitis vinifera*	LN-RI, LN-Cas, LN-ANG, LN-KTST, SD-ITRI-8-2,	China
2018, 2018, 2018	LC746709, LC815109, LC815111	*Vitis* spp.,*Vitis* spp.,*Vitis* spp.	g1-C194, g8-C2609, g13-C130	Japan
2022	LC718555	*Vitis* sp.	KG	Korea
2015, 2015,	KU362270, KU362275	*Vitis vinifera, Vitis vinifera*	81, 129,	Turkey
2021, 2021, 2021	PQ521015, PQ521016, PQ521017	*Vitis vinifera, Vitis vinifera, Vitis vinifera*	510, 511, 515	Russia
2016	KY821089	*Vitis vinifera*	SL37-1	Pakistan
2011, 2011	KC567952, KC567953	*Vitis vinifera, Vitis vinifera*	7 sequence variant 1, 7 sequence variant 3	Portugal
2010, 2015, 2012, 2018, 2010	NC_016509, MH545961, MH807218, MZ344577, JQ023131	–, *Vitis vinifera* , *Vitis vinifera*, grapevine, –	1050, ON84-15, 12G456, 18GVA002b, 1050	Canada
2012, 2013, –, –	KU674796, KU674797, JF811834, JF811844	*Vitis vinifera, Vitis vinifera, Vitis vinifera, Vitis vinifera*	WA-CH, WA-PN, CA11, CA20	USA
2019, 2019, 2019, 2019	MT953192, MT953193, MT953194, MT953195	*Vitis vinifera, Vitis vinifera, Vitis vinifera, Vitis vinifera*	RSA-03-06, RSA-06-08, RSA-12-04, RSA-18-02	South Africa
2012, 2012	MG925331, MG925332	*Vitis vinifera, Vitis vinifera*	P70, P70	France
–,	KY827404	*Vitis vinifera*	Ti23	Switzerland
2017, 2018, 2019	OQ029645, OQ029678, OQ029646	*Vitis vinifera, Vitis vinifera, Vitis vinifera*	SK809, AUTH63, Pin1,	Spain
2016	OP718744	*Vitis* sp.	NG66	Nigeria

^*a*^ New Iranian isolate in this research showed in Bold.

^*b*^ “–” indicates that there are no information.

**TABLE 2:** Properties of new isolates of GLRaV-1 identified in this study from Iran

Location	GPS coordinates	Number	ELISA^*a*^	RT-PCR^*a*^	Host	Accession number	Collection date
West Azerbaijan	37.7595°N, 45.0000°E	68	14	A24	*Vitis vinifera*	PP213148	2023
Qazvin	36.2795°N, 50.0046°E	34	10	–^*b*^	–	–	–
Fars	29.1044°N, 53.0459°E	28	7	–	–	–	–
Kohgiluyeh and Boyer-Ahmad	30.7246°N, 50.8456°E	136	21	KG76, KG74, KG64, KG56, KG31, KG75	*Vitis vinifera, Vitis vinifera, Vitis vinifera, Vitis vinifera, Vitis vinifera, Vitis vinifera*	PP213141, PP213142, PP213143, PP213144, PP213145, PP213146, PP213147	2023, 2022, 2023, 2023, 2022, 2023
Khorasan Razavi	35.1020°N, 59.1042°E	59	7	K78, K27	*Vitis vinifera, Vitis vinifera*	PP213146, PP213147	2023, 2023
**Total Samples**		**325^*c*^**	**59**	**9**			

^*a*^ Positive samples.

^*b*^ “–” indicates that there are no information.

^*c*^ Bold values indicates the total os samples.

### Serological test and RNA extraction and RT-PCR

Double-antibody sandwich enzyme-linked immunosorbent assay (DAS-ELISA) was performed using virus-specific antibodies against GLRaV-1 and GLRaV-3 (Bioreba AG, Reinach, Switzerland) according to the manufacturer’s instructions. Leaf samples (0.5 g) were ground in extraction buffer (PBST containing 2% PVP-40 and 0.05% Tween-20) at a ratio of 1:10 (wt/vol) using a chilled mortar and pestle. Microtiter plates were coated with 200 µL of IgG antibody diluted 1:200 in carbonate-bicarbonate buffer (pH 9.6) and incubated overnight at 4°C. After three washes with PBST, 200 µL of plant extract was added to each well and incubated for 2 h at 37°C. Plates were washed again before adding 200 µL of alkaline phosphatase-conjugated antibody diluted 1:200 in PBST, followed by incubation for 2 h at 37°C. After a final wash, 200 µL of p-nitrophenyl phosphate (pNPP, 1 mg/mL in diethanolamine buffer, pH 9.8) was added, and the reaction was developed for 60 min at room temperature in the dark. Absorbance was measured at 405 nm using a Microplate Reader. Samples with absorbance values exceeding the mean of healthy controls by at least three times the standard deviation were considered positive.

Total RNA was extracted from symptomatic grapevine leaf tissues using the GeneAll Plant RNA Extraction Kit (GeneAll Biotechnology, South Korea) according to the manufacturer’s protocol. The quantity and purity of the extracted RNA were assessed using a NanoDrop 2000 spectrophotometer (Thermo Scientific, USA). The RNA concentration and absorbance ratios at 260/280 nm were recorded to evaluate RNA quality. Samples with A260/A280 ratios between 1.8 and 2.0 were considered suitable for downstream applications.

### cDNA synthesis

Reverse transcription (RT) was carried out using the Easy cDNA Synthesis Kit (Parstous, Iran) following the manufacturer’s instructions. The total reaction volume was 10 µL, and the thermal cycling conditions for RT included 25°C for 10 min primer annealing, 47°C for 60 min reverse transcriptase enzyme activity, and 85°C for 5 min enzyme inactivation. The reaction was performed in a T100 Thermal Cycler (Bio-Rad, USA).

### Polymerase chain reaction amplification

The incidence of GLRaV-1 was determined using a pair of primers (22), to amplify a 232 bp DNA fragment. Subsequently, PCR amplification of the GLRaV-1 coat protein (CP) gene was performed using specific primers (5, 19): Forward primer (GLRaV-1 CP/F): CGCGCTTGCAGAGTTTAAGTGGTT and Reverse primer (GLRaV-1 CP/R): TCCGTGCTGCATTGCAACTTTCTC. PCRs were performed in a total volume of 25 µL, consisting of 1 µL cDNA template, 12.5 µL 2 × PCR Master Mix (Ampliqon, Denmark), 1 µL of each primer (10 µM), and 9.5 µL nuclease-free water. The thermal cycling conditions for PCR were as follows: initial denaturation at 94°C for 3 min, 40 cycles of: 94°C for 30 s (denaturation), 58°C for 30 s (annealing), 72°C for 45 s (extension), final extension at 72°C for 5 min

PCR products were analyzed by 1% agarose gel electrophoresis in 1 × TAE buffer and visualized under UV light after staining with GelRed (Biotium, USA). Amplified products were subsequently purified and sent for sequencing to assess the genetic diversity of GLRaV-1 isolates.

### Phylogenetic analysis

Phylogenetic analyses of 24 complete GLRaV-1 sequences available in the GenBank database worldwide were carried out using NCBI-BLAST (http://www.ncbi.nlm.nih.gov/BLAST/) (Table 1). Sequence alignment was performed with MEGA 11 software (23), utilizing the Clustal W program (24). In addition, the obtained nucleotide sequences of PCR products were compared with reference sequences available in the NCBI GenBank database using the BLAST algorithm (http://www.ncbi.nlm.nih.gov/BLAST/). The nine newly obtained nucleotide sequences were aligned with 61 previously reported GLRaV-1 sequences available in the NCBI GenBank database (Table 1). The complete genome alignment was 17,649 nt in length after trimming. For the coat protein (CP) data set, the same 9 new isolates and 61 previously reported isolates from different geographical origins were included, resulting in an alignment length of 732 nt. Multiple sequence alignment was performed using MEGA 11 software (25) and the ClustalW algorithm (24). After alignment, necessary edits were applied for further analysis. The evolutionary distances were computed using the Jukes-Cantor model. The best-fit nucleotide substitution model for phylogenetic tree construction was selected using MEGA 11. Phylogenetic relationships among isolates were inferred using the Neighbor-Joining method (26) with the Tamura 3-parameter model (25), implemented in MEGA 11. Bootstrap resampling with 1,000 replicates was applied to assess the robustness of the phylogenetic relationships, and branches with bootstrap values below 50% were collapsed to improve clarity. The phylogenetic tree was generated based on the coat protein (CP) and complete genome sequences. Recombinant groups and isolates with <0.5% genetic distance were omitted to enhance the resolution of clustering patterns. The final phylogenetic tree provided insights into the genetic diversity of GLRaV-1 isolates from Iran in comparison to global isolates, and detailed information on Iranian isolates is presented in Table 1. Furthermore, a pairwise nucleotide sequence identity matrix was generated using MEGA 11 to quantify the genetic similarity among the studied isolates.

### Recombination analysis and phylogeny

To detect potential recombination events and phylogenetic anomalies, the Recombination Detection Program 4 (RDP4) was used with its comprehensive suite of analytical tools, including RDP, Chimaera, MaxChi, Bootscan, Siscan, GENECONV, and 3Seq. The analysis was conducted using default parameters, applying a *P*-value threshold of 0.05 to identify significant recombination signals. Any anomalies that were supported by fewer than five of these detection methods and had a Bonferroni-corrected *P*-value below 0.05 were excluded from further consideration. To validate the parent/recombinant relationships of the two recombinant strain clusters, the sequence alignments were divided into regions that either contained or excluded the recombinant segments.

### Population genetic parameters

The genetic differentiation of the coat protein (CP) gene and complete genomes across diverse populations was analyzed using DnaSP v6.10.01. Several genetic parameters were examined, including haplotype diversity (Hd), the number of polymorphic sites (*S*), total number of mutations (*η*), average nucleotide differences (*k*), pairwise nucleotide diversity (*π*), and the ratio of non-synonymous to synonymous substitutions (d*N*/d*S*). To assess neutral selection, Tajima’s D (27) and Fu and Li’s *D* and *F* tests (28) were applied. To investigate population differentiation, statistical tests such as KS, Z, Snn, and FST (fixation index) were performed using 1,000 replicates (29). The pairwise nucleotide sequence identity was calculated in MEGA 11 to evaluate genetic similarity between isolates.

### Molecular dating analysis

Divergence times of the GLRaV-1 population along with four closely related *Ampelovirus* species, including *grapevine leafroll-associated virus-3* (GLRaV-3), *grapevine leafroll-associated virus-13* (GLRaV-13), *grapevine leafroll-associated virus-4* (GLRaV-4), and *little cherry virus 2* (LChV2) was estimated (Table 2). Additionally, grapevine leafroll-associated virus-2 (GLRaV-2), a *Closterovirus* species, was used as an outgroup for comparative analysis. To estimate divergence times, internal node ages were analyzed, and a TimeTree was reconstructed using the RelTime-ML computational method under the Tamura 3-parameter model (30) within MEGA 11 software. The time to the most recent common ancestor (TMRCA) was calculated using the default calibration method (31). A discrete Gamma distribution with five categories (+G, parameter = 2.2907) was applied to model evolutionary rate variation across different sites, while 8.65% of sites were classified as evolutionarily invariable ([+I]). This analysis included 35 nucleotide sequences, incorporating *1st, 2nd*, and *3rd* codon positions, as well as noncoding regions, resulting in a final data set of 6,302 positions. The RelTime method estimated divergence times exclusively for the ingroup clade, as it does not assume that the evolutionary rates of the ingroup apply to the outgroup. Therefore, divergence times for outgroup nodes were not calculated. All estimated divergence times are relative since no external calibrations were applied.

## RESULTS

### Field observation

Observations from vineyard surveys in West Azerbaijan, Fars, Qazvin, Kohgiluyeh and Boyer-Ahmad, and Razavi Khorasan provinces (2019–2023) confirmed the presence of symptoms consistent with grapevine leafroll disease, including leaf reddening, chlorotic spotting, curling, deformation, and reduced leaf size. In some vineyards, leaf curling was particularly prevalent. Table 2 provides the detailed mapping of newly identified GLRaV-1 haplotypes to their respective collection sites, GPS coordinates, and collection dates. Other plant diseases, such as powdery mildew, were also observed in several vineyards.

### ELISA detection

Out of the 325 grapevine samples collected across West Azerbaijan, Fars, Qazvin, Kohgiluyeh and Boyer-Ahmad, and Razavi Khorasan provinces (2019–2023), all samples were tested for GLRaV-1+3 infection using DAS-ELISA with virus-specific antibodies (Bioreba AG, Switzerland) according to the manufacturer’s instructions. Among these, 59 samples (18.2%) tested positive for GLRaV-1, confirming the presence of the virus in multiple provinces. The highest infection rate was observed in Kohgiluyeh and Boyer-Ahmad (21/136 samples), followed by West Azerbaijan (14/68 samples, Fars (7/280 samples), Qazvin (10/34 samples), and Razavi Khorasan (7/59 samples).

### PCR amplification

PCR reactions were performed using two specific forward and reverse primer pairs (R) and (F) targeting the coat protein gene of GLRaV-1, the virus associated with grapevine leafroll disease. This process successfully amplified and detected DNA fragments of 734 base pairs. Nine positive samples were sequenced based on regions and symptoms, and the sequences were uploaded to GenBank NCBI, under the accession numbers PP213141 to PP213148 and PP182259. The results of recombinant event analysis using the RDP4 program, which includes several recombinant detection algorithms such as RDP, Chimaera, Maxchi, Bootscan, GENECONV, and SISCAN, revealed the presence of recombinant strains among the studied isolates. Among the newly identified isolates in this study, isolate PP182259, detected in the Kohgiluyeh and Boyer-Ahmad province, was found to be a recombinant virus associated with grapevine leafroll associated virus-1, with mutations having occurred. In other words, the recombination regions in this isolate were found to be swapped. The parental strain of the newly identified isolate PP213143 from Kohgiluyeh and Boyer-Ahmad is of unknown origin, and recombinant events in this isolate were detected as positive using three recombinant detection methods (M, C, T), increasing the likelihood of true recombination. Consequently, isolate PP182259, identified as a recombinant sequence, should be excluded from phylogenetic analysis to avoid distortion of the results (32).

### Phylogenetic analysis

**Fig 1:**
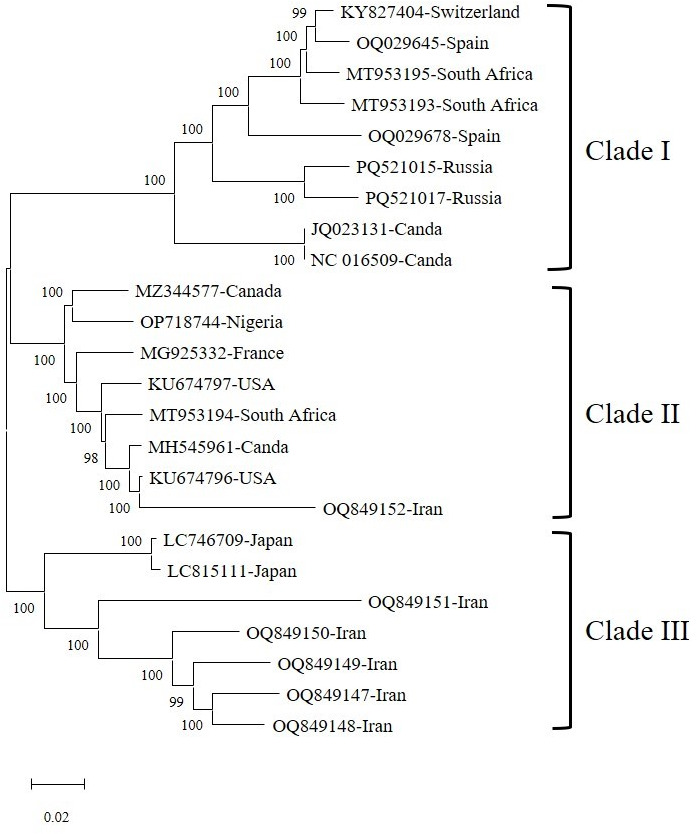
Phylogenetic Tree of GLRaV-1 isolates/strains reconstructed based on complete genome sequences by the Neighbor Joining (NJ) method based on Tamura 3-parameter’s model with Gamma Distributed(G) and Invariant sites (I) (T92 + G + I) in MEGA 11. Bootstrap values over 50% are given at the nodes.

The phylogenetic tree constructed from the analyzed sequences based on the complete genome revealed three distinct major clades, each strongly supported by high bootstrap values (98–100), indicating robust phylogenetic relationships and genetic differentiation among isolates (Fig. 1). Clade I, the first major clade consists of isolates such as KY827404, OQ029645, MT953195, MT953193, OQ029678, PQ521015, and PQ521017, all forming a well-supported monophyletic group with a bootstrap value of 100 (Fig. 1; Table 1). Clade II, the second major clade encompasses a diverse set of isolates, including MZ344577, OP718744, MG925332, KU674797, MT953194, MH545961, and KU674796 (Fig. 1; Table 1).

**Fig 2:**
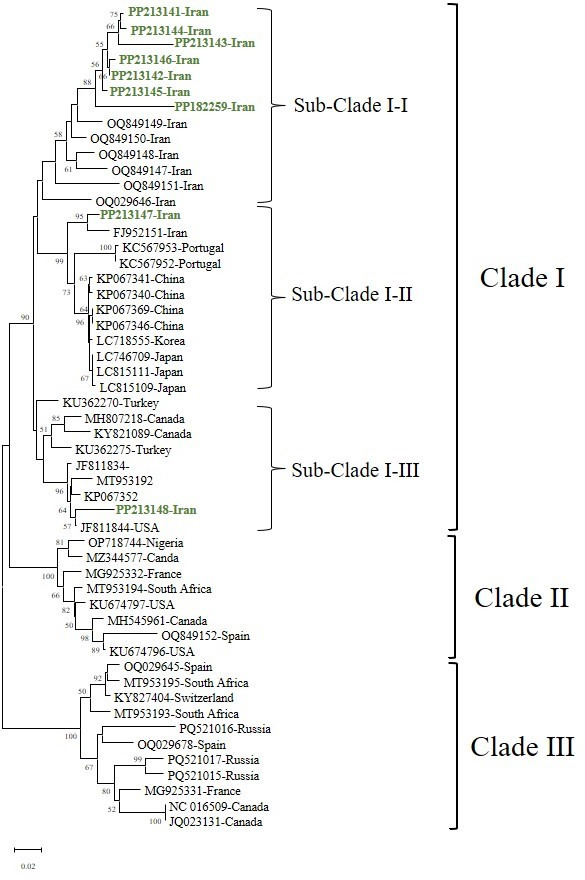
Phylogenetic Tree of GLRaV-1 isolates/strains reconstructed based on complete coat protein sequences by the Neighbor Joining (NJ) method based on Tamura 3-parameter’s model with Gamma Distributed(G) and Invariant sites (I) (T92 + G + I) in MEGA 11. Bootstrap values over 50% are given at the nodes. The new Iranian isolates have been shown with green bold.

**Fig 3:**
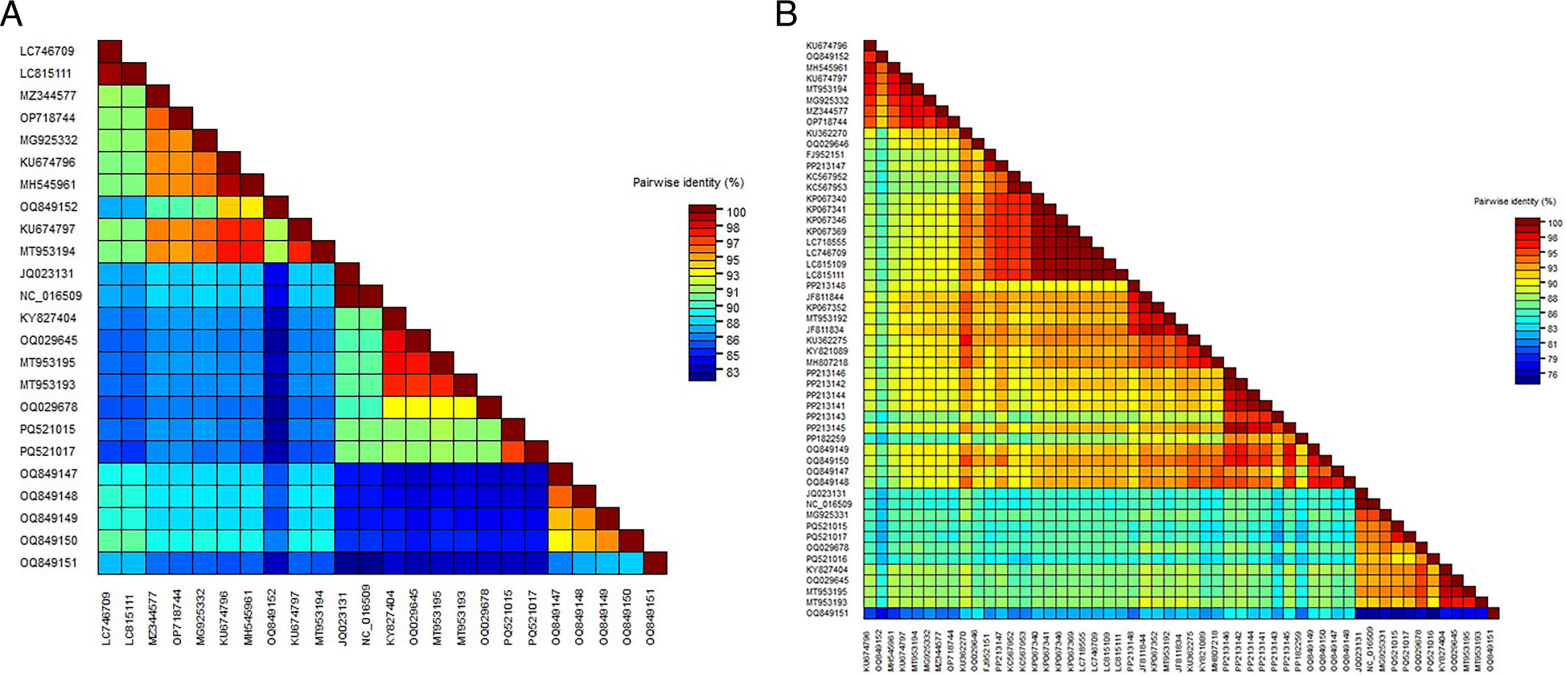
A pairwise sequence comparison of the complete genome sequence of GLRaV-1 isolates (**A**) and the coat protein (cp) gene (**B**) was conducted using the Sequence Demarcation Tool (SDT). The pairwise sequence identities (%) between the isolates are visually represented with color-coded keys. Each color corresponds to a specific percentage of identity between two sequences, offering a clear illustration of the genetic relatedness and diversity among the GLRaV-1 isolates.

The phylogenetic analysis of GLRaV-1 based on the CP gene sequences revealed three well-supported clades, indicating significant genetic diversity among the isolates (Fig. 2; Table 1). The newly identified Iranian isolates (bold accession numbers) clustered within different groups, suggesting multiple evolutionary origins of GLRaV-1 in this region (Fig. 2; Table 1). The first major clade (Clade I) contains the Iranian isolates PP213141– PP213148 and PP182259, which are closely related and form a distinct monophyletic group (Fig. 2). While these isolates do not all belong to the same subgroup, their placement within Clade I suggests they share a broader evolutionary lineage. This pattern may reflect multiple introductions, local divergence, or incomplete sampling from other regions (Fig. 2). All Iranian isolates were grouped within the first major clade (Clade I), which contains several sub-clades. Within Clade I, isolate PP213147 grouped in a distinct sub-clade, isolate PP213148 is placed in another separate sub-clade, and the remaining Iranian isolates are grouped in a different sub-clade within Clade I (Fig. 2; Table 1). This sub-clade structure indicates that, although all Iranian isolates belong to the same major clade, they exhibit genetic differentiation that may reflect localized divergence or other evolutionary processes (Fig. 2 and 3; Table 1).

### Genetic diversity and population structure analysis

Genetic diversity analysis of the complete genome sequences revealed substantial variation among the three clades. Haplotype diversity was high in all clades, with Clades II and III showing Hd = 1 and Clade I slightly lower (Hd = 0.972). Polymorphic sites (*S*), nucleotide diversity (*π*), and average nucleotide differences (*k*) were highest in Clade III (*S* = 3,562, *π* = 0.09334, *k* = 1,523.429), indicating greater genetic variation, followed by Clade I and Clade II. Selection pressure analysis (*ω* = d*N*/d*S*) showed values below 1 for all clades (Clade I = 0.49, Clade II = 0.54, Clade III = 0.58), consistent with predominant purifying selection, while Clade III had the highest d*N* (0.079) and d*S* (0.135), suggesting stronger evolutionary diversification compared to the more conserved Clade II (d*N* = 0.043, d*S* = 0.079) (Table 3).

**TABLE 3:** Genetic diversity and polymorphism analyses of GLRaV-1 complete genome sequences^*a*^

Population	*N*	*h*	Hd	*S*	*η*	*k*	*π*	d*S*	d*N*	*ω*
Complete genome	24	23	0.996	6,539	8,278	1,842.837	0.11704	0.180	0.096	0.53
Phylogroups (complete genome)										
Clade I	9	9	0.972	3,411	3,724	1,305.861	0.07468	0.122	0.060	0.49
Clade II	8	8	1	2,807	2,999	912.321	0.05202	0.079	0.043	0.54
Clade III	7	7	1	3,562	3,891	1,523.429	0.09334	0.135	0.079	0.58
Phylogroups (coat protein)										
Clade I	34	32	0.996	135	159	28.41	0.07120	0.2239	0.0242	0.10
Clade II	8	8	1	53	60	19	0.04222	0.1042	0.02238	0.21
Clade III	11	10	0.982	83	94	28.29	0.06287	0.2208	0.01433	0.06

^*a*^ *N*, number of isolates; *h*, number of haplotypes; Hd, haplotype diversity; *S*, number of variable sites; *η*, total number of mutations; *k*, average number of nucleotide differences between sequences; *π*, nucleotide diversity (per site); d*N*, non-synonymous nucleotide diversity; d*S*, synonymous nucleotide diversity; *ω*, d*N*/d*S*.

Population genetic analysis of GLRaV-1 based on CP gene sequences revealed high genetic diversity and strong purifying selection across three clades. Clade I (*N* = 34) exhibited the highest nucleotide diversity (*π* = 0.0712), average nucleotide differences (*k* = 28.41), segregating sites (*S* = 135), and total mutations (*η* = 159), with Hd = 0.996 and *ω* = 0.108, indicating a highly diverse yet evolutionarily constrained population (Tables 1 and 4). Clade II (*N* = 8) showed lower nucleotide diversity (*π* = 0.0422) and fewer mutations (*S* = 53, *η* = 60), but the highest haplotype diversity (Hd = 1.00) and *ω* = 0.21, suggesting potential relaxed purifying or episodic positive selection in certain CP regions (Table 3). Clade III (*N* = 11) displayed moderate diversity (Hd = 0.982, *π* = 0.0628, *S* = 83, *η* = 94) with the lowest *ω* (0.064), reflecting strong purifying selection and evolutionary conservation of the CP gene (Table 3). Overall, *ω* values < 1 in all clades indicate that purifying selection predominates, removing deleterious amino acid changes, while differences among clades suggest ongoing evolutionary adaptation influenced by host interactions and vector-mediated transmission. Clade I represents a highly diverse lineage comprising all newly characterized Iranian isolates, highlighting the importance of continuous genetic surveillance for effective virus management.

**TABLE 4:** Results from demography test statistics between sequences of complete genome

Population	Fu and Li’s *D^a^*	Fu and Li’s *F^a^*	Tajima’s *D*
Complete genome			
Phylogroup			
Clade 1	0.03757 ns^*b*^	−0.03517 ns	−0.24549
Clade 2	−1.18577 ns	−1.31943 ns	−1.16266
Clade 3	−0.07682 ns	−0.12613 ns	−0.24048
CP			
Phylogroup			
Clade 1	−1.44256 ns	−1.53954 ns	−1.01977
Clade 2	−0.84671 ns	−1.31943 ns	−0.96633
Clade 3	−0.36167 ns	−0.47084 ns	−0.56722

^*a*^ 0.01 < *P* value > 0.05; 0.001 < *P* value > 0.01; *P* value < 0.001.

^*b*^ ns, not significant.

### Population differentiation and genetic structure analysis

Analysis of neutrality and population differentiation of GLRaV-1 revealed insights into evolutionary dynamics among the three clades. Tajima’s *D* values were negative in all clades, indicating an excess of low-frequency polymorphisms consistent with past population expansion or purifying selection, with Clade I showing the most negative value (−1.44256) though none were statistically significant (Table 4). Fu and Li’s *D* and F** values showed similar trends, with Clade I and II exhibiting more negative values, suggesting historical expansion or selective constraints, while Clade III appeared relatively stable (Table 4). Genetic differentiation analysis based on the complete genome indicated substantial divergence among clades, with the highest FST between Clade I and II (0.547), followed by Clade I/III (0.457) and Clade II/III (0.389), reflecting limited gene flow (Table 5). Additional tests (KS, KST, Z, Snn, FST) confirmed significant population structure, with KS and Z values high for all comparisons (Clade I/II: KS = 6.79, *Z* = 3.19; Clade I/III: KS = 6.94, *Z* = 3.11; Clade II/III: KS = 6.84, *Z* = 3.12) and all *S*_nn_ = 1.00, with highly significant *P*-values (*P* = 0.0000), supporting strong genetic differentiation and limited gene flow among the clades (Table 5).

**TABLE 5:** Genetic differentiation estimates for lineage, based on complete genome and CP gene sequences comparison^*a*^^,*b*^

Comparison	*^b^K*_S*_	*^b^K*_ST*_	*P* value	^c^Z*	*P* value	*S*_nn_	*P* value	*^c^F*_ST_
Complete genome								
Phylogroup								
Clade1/Clade2	6.79	0.071	0.000	3.19	0.00	1.00	0.000	0.547
Clade 1/Clade3	6.94	0.061	0.000	3.11	0.00	1.00	0.000	0.457
Clade 2/Clade3	6.84	0.049	0.000	3.12	0.00	1.00	0.000	0.389
CP								
Phylogroup								
Clade1/Clade2	3.19	0.06	0.000	5.34	0.00	1.00	0.000	0.453
Clade 1/Clade3	3.22	0.08	0.000	5.40	0.00	1.00	0.000	0.521
Clade 2/Clade3	3.12	0.14	0.000	3.41	0.00	1.00	0.000	0.622

^*a*^ 0.01 < *P* value > 0.05; 0.001 < *P* value > 0.01; *P* value < 0.001.

^*b*^ *K*_S_*, *K*_ST_*, *Z,* and *S*_nn_ are test statistics of genetic differentiation diversity.

^*c*^ *F*_ST_, coefficient of gene differentiation, which measures inter-population diversity.

All pairwise comparisons based on the CP sequences yielded highly significant *P*-values (*P* = 0.000) across multiple differentiation metrics, indicating strong genetic divergence between the clades. KS and KST values indicated significant differentiation in sequence composition, with the highest observed between Clade 1 and Clade 3 (KS = 3.22, KST = 0.08), followed by Clade 1 vs Clade 2 (KS = 3.19, KST = 0.06). The *Z* statistic was consistently high across all comparisons, particularly between Clade 1 and Clade 3 (*Z* = 5.40), indicating strong genetic separation. The *S*_nn_ test yielded values of 1.00 for all comparisons, confirming nearly complete genetic differentiation, where sequences within each clade are more closely related to each other than to sequences in other clades. FST values were highest between Clade 2 and Clade 3 (FST = 0.622), followed by Clade 1 vs. Clade 3 (FST = 0.521) and Clade 1 vs Clade 2 (FST = 0.453), demonstrating substantial genetic divergence likely influenced by distinct evolutionary pressures.

The strong genetic differentiation among the three GLRaV-1 clades indicates limited gene flow and independent evolutionary trajectories. High FST values suggest adaptation to different host populations or vector transmission dynamics, reinforcing distinct phylogenetic clustering. Clade 1, which includes all Iranian isolates, shows strong divergence from Clades 2 and 3, reflecting region-specific evolution, while the differentiation between Clade 2 and Clade 3 implies genetically distinct populations, possibly due to long-term geographical isolation or selective pressures. Demographic tests indicate that the GLRaV-1 population, particularly Clade 1, has likely undergone historical expansion, potentially linked to the spread of infected grapevine cultivars, vector activity, or ecological changes facilitating viral dissemination. Low d*N*/d*S* ratios suggest that purifying selection predominates, maintaining structural and functional integrity of the coat protein essential for viral transmission. Clade-specific patterns show that Clade 1 exhibits the strongest signals of demographic expansion, Clade 2 shows moderate expansion, and Clade 3 appears relatively stable, indicating long-term population equilibrium.

### Sliding-window analysis of polymorphism

**Fig 4:**
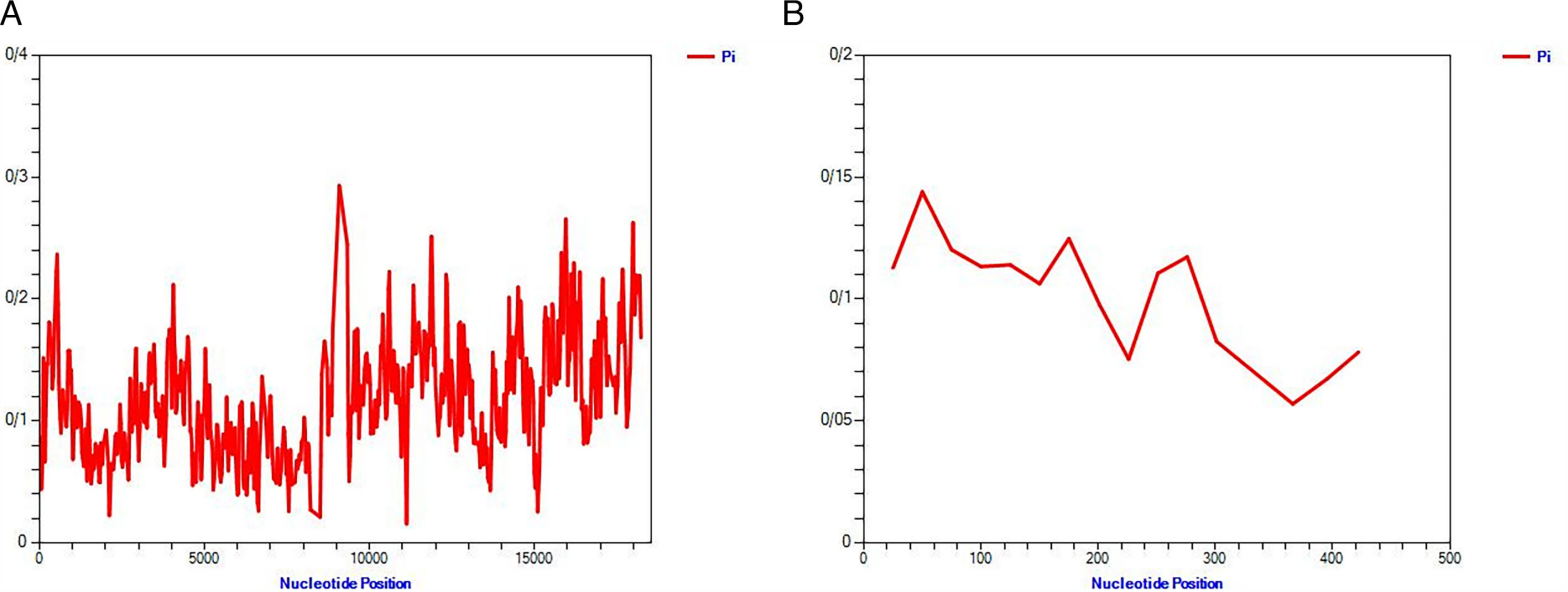
Sliding-window plot of polymorphism and divergence levels of complete genome sequence of GLRaV-1 isolates (**A**) and the coat protein (cp) gene (**B**).

A sliding-window analysis of nucleotide diversity (*π*) across the complete genome of GLRaV-1 revealed fluctuations in genetic variability, with distinct peaks and valleys indicating regions of high and low polymorphism (Fig. 4). Overall, *π* remained relatively low across most of the genome, with moderate variability in the 5′ and 3′ regions, suggesting conserved sequences likely due to functional or structural constraints. Notably, a peak around nucleotide position 10,000 indicates a region of heightened polymorphism, potentially representing a recombination hotspot, positive selection, or relaxed evolutionary constraints. Beyond position 15,000, diversity levels show an upward trend, highlighting another region with increased genetic variation (Fig. 4).

A sliding-window analysis of nucleotide diversity (*π*) across the *GLRaV-1* CP gene revealed fluctuations in sequence variability along the gene (Fig. 4). Nucleotide diversity ranged from approximately 0.05 to 0.18, with the highest variability observed between 50 and 150 bp, suggesting potential hotspots of polymorphism, while a conserved region was detected between 250 and 350 bp, likely reflecting functional or structural constraints. A slight increase in diversity after 400 bp indicates minor accumulation of polymorphisms. High-variability regions may be under selective pressure related to host adaptation or immune evasion, whereas low-diversity regions likely maintain essential viral structure and replication efficiency. Overall, this analysis highlights a balance between evolutionary constraints and adaptation, providing insights for molecular diagnostics, antiviral strategies, and resistance breeding programs (Fig. 4).

### Time to the most recent common ancestor estimation

**Fig 5:**
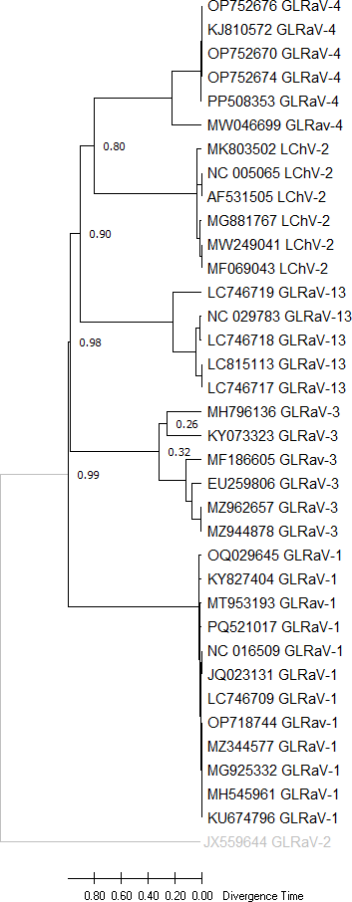
Timetree analysis and divergence time estimation of GLRaV-1 and related *Ampelovirus* species. The TimeTree was constructed using the RelTime-ML method under the Tamura 3-parameter model (30) in MEGA 11 software. The analysis included GLRaV-1 and four closely related Ampelovirus species: GLRaV-3, GLRaV-13, GLRaV-4, and LChV-2, with GLRaV-2 (Closterovirus species) used as an outgroup. Divergence times were estimated based on internal node ages with the time to the most recent common ancestor (TMRCA) calculated using the default calibration method (31). A discrete Gamma distribution (+G, parameter = 2.2907) was applied to model rate variation across sites, and 8.65% of sites were considered invariable (+I). The analysis involved 35 nucleotide sequences, including codon positions 1st, 2nd, 3rd, and noncoding regions, with a total of 6,302 positions. Divergence times for outgroup nodes were not calculated. All estimates are relative, as no external calibrations were applied.

A time-calibrated phylogenetic tree of *Ampelovirus* members was reconstructed using the RelTime method in MEGA 11, based on complete or near-complete genome sequences, with GLRaV-2 (JX559644) as the outgroup (Fig. 5; Table 2). The analysis resolved five major monophyletic clades corresponding to GLRaV-1, GLRaV-3, GLRaV-4, GLRaV-13, and LChV-2. The GLRaV-1 clade, including isolates such as OQ029645, KY827404, NC_016509, and MH545961, exhibited a relative divergence time of ~0.20, indicating recent diversification (Tables 6 and 7). In contrast, GLRaV-3 and GLRaV-13 showed deeper divergence (~0.26–0.32 and ~0.30, respectively), while GLRaV-4 formed a compact cluster with shallow divergence (≤ 0.10), suggesting low sequence variability or recent expansion. LChV-2 diverged at ~0.45, and the most basal split occurred between GLRaV-2 and all other Ampelovirus taxa (~0.80). Bootstrap support values were generally high (>0.90), confirming the robustness of the inferred phylogeny and supporting current taxonomic groupings within the genus *Ampelovirus*.

**TABLE 6:** Estimated GLRaV-1 TMRCA based on the ratio of the patristic distances within the four ampelovirus complete genome sequence Maximum-Likelihood tree

Species	Mean patristic distance	Ratios of GLRaV-1 and other patristic distances
GLRaV-1	0.20	1
GLRaV-4	0.10	0.50
GLRaV-13	0.30	1.50
GLRaV-3	0.32	1.60
LChV-2	0.45	2.25
GLRaV-2 (outgroup)	0.80	4.00

**TABLE 7:** Different isolates that used for TMRCA analysis

Collection Date	Accession number	Host	Isolates/strains	Country
2019, 2019, 2019, 2022,	OP752676, OP752670, OP752674, PP508353	*Vitis vinifera, Vitis vinifera, Vitis vinifera, Vitis vinifera*	LC5_N5, LC14_N6, WIL6_N6, 7_16_GLRaV_4	Australia
2011, 2017	KJ810572, OQ029645	*Vitis vinifera, Vitis vinifera*	Man086, SK809	Spain
2019, -, 2019	MW046699, EU259806, MT953193	*Vitis vinifera, –, Vitis vinifera*	RSA-15-14_Hebron, GP18, RSA-06-08_Blauer_Limberger	South Africa
2016, 2017	MK803502, MW249041	*Prunus avium, Prunus serrulata*	GBVC_LChV2_100 BEREF16, GBVC_LChV-2_201	Belgium
-, 2010-12-16, 2017, 2021, 2021, 2010, 2016, 2012,	NC_005065, NC_029783, MH796136, MZ962657, MZ944878, NC_016509, OP718744, KU674796	*–^a^, Vitis vinifera, Vitis vinifera, Vitis aestivalis, Vitis aestivalis, Vitis* sp*, Vitis* sp*., Vitis vinifera*	USA6b, a177, GLRaV3-ID45, 170, C20, 1050, NG66, WA-CH	USA
–,	AF531505	–,	USA6b	Germany
2016	MG881767	–,	LChV-2-TA	China
2014	MF069043	Cherry	Rube 74	Czech Republic
2018-10, 2018-10, 2018-7, 2018-7, 2018-07	LC746719, LC746718, LC815113, LC746717, LC746709	*Vitis* spp.*, Vitis* spp.*, Vitis* spp.*, Vitis* spp*., Vitis* spp.	g12-C21, g11-C1624, g2-C698, g9-C618, g1-C194	Japan
2014, 2010, 2018, 2014, 2011	KY073323, JQ023131, MZ344577, MH545961, JX559644	*Vitis vinifera, -, grapevine, grapevine, Vitis vinifera*	8415A, 1050, 18GVA002b, ON84-15, 3138-07	Canada
2015	MF186605	*Nicotiana benthamiana*	GLRaV-3-I-LR101	Croatia
–,	KY827404	Grapevine	Ti23	Switzerland
2021	PQ521017	*Vitis vinifera*	515	Russia
2012	MG925332	*Vitis vinifera*	P70	France

^*a*^ “–” indicates that there are no information.

To estimate the evolutionary age of GLRaV-1, the time to its most recent common ancestor (TMRCA) was calculated using the RelTime-derived relative divergence (*D* = 0.20) and a reported Closteroviridae substitution rate (*μ* = 1 × 10⁻⁴ substitutions/site/year), suggesting that GLRaV-1 likely emerged approximately 1,000 years ago (~1,025 C.E. CE), potentially coinciding with historical grapevine domestication and viticulture expansion. GLRaV-1 exhibited the lowest mean patristic distance (0.20), serving as a baseline for comparison. GLRaV-2, used as the outgroup, had the highest mean patristic distance (0.80), indicating greater evolutionary divergence from GLRaV-1. Among other strains, GLRaV-4 showed the smallest distance (0.10), while GLRaV-13 (0.30) and GLRaV-3 (0.32) were slightly more distant, and LChV-2 had the largest divergence (0.45), with ratios of 1.50, 1.60, and 2.25 relative to GLRaV-1, respectively (Table 6). These results indicate varying levels of genetic divergence among the strains, with GLRaV-1 being most closely related to the other lineages, while GLRaV-2 represents a more distant evolutionary lineage.

## DISCUSSION

Ampeloviruses, particularly GLRaV-1, represent a major challenge to global viticulture due to their wide distribution, significant economic impact, symptom overlap with nutrient deficiencies, and other important factors such as vector transmission and vegetative propagation. The precise identification of GLRaV-1 and the study of its genetic variability are essential for effective disease management and epidemiological surveillance. Understanding viral population structure and evolutionary dynamics also aids in tracing epidemic pathways and anticipating the emergence of novel variants (33). In this study, symptomatic grapevine samples were collected from five major grape-growing provinces in Iran. Consistent with previous reports, visual symptoms varied between red and white cultivars and changed seasonally. Molecular detection confirmed GLRaV-1 in several regions, with Khorasan Razavi showing the highest infection rate, while no virus was detected in Fars and Qazvin, possibly due to primer mismatch from sequence divergence in the targeted region. Phylogenetic analysis of coat protein (CP) and complete genome sequences revealed three major clades, indicating a complex phylogeographic structure. Several Iranian isolates formed a distinct lineage, highlighting substantial regional diversity and the likelihood of localized viral evolution. This pattern is consistent with earlier studies reporting limited gene flow and geographically structured GLRaV-1 populations (19, 34, 35).

Genetic diversity analysis based on the complete genome sequences showed that Clade III exhibited the highest nucleotide and haplotype diversity, as well as signs of diversifying selection (*ω* = 0.58). In contrast, Clade I was under stronger purifying selection (*ω* = 0.49), reflecting evolutionary constraints on essential viral genes. High *F*_ST_ values between clades (0.389–0.547) confirmed significant genetic differentiation and low inter-population gene flow. Statistical measures of genetic differentiation (*K*_S_, *Z*, *S*_nn_, and *F*_ST_) confirmed the presence of three genetically isolated GLRaV-1 populations. These clades appear to have followed distinct evolutionary trajectories, shaped by geographic separation, host genotype preferences, and vector specificity. Recombination was limited, reinforcing the clade structure and supporting the observed patterns of genetic partitioning.

Population genetic parameters derived from CP sequences further corroborated these trends. The Iranian group displayed the highest mutation rate, number of polymorphic sites, and mean nucleotide divergence. Such variability may increase the likelihood of recombination and contribute to rapid virus evolution. In fact, one isolate (PP182259) from Kohgiluyeh and Boyer-Ahmad was identified as a recombinant, highlighting the dynamic nature of GLRaV-1 evolution in Iran. This finding is consistent with the known high mutation and recombination rates in positive-sense RNA viruses (36, 37).

Neutrality tests (Tajima’s *D*, Fu and Li’s D*/F*) yielded negative values across all Iranian clades, indicative of recent population expansion or bottlenecks. These may be attributed to vector transmission, propagation practices, or environmental stress. In contrast, positive neutrality values in European and East Asian populations point to evolutionary stability and reduced selection pressure.

Sliding-window analysis identified nucleotide diversity hotspots within the GLRaV-1 genome, indicating regions potentially under selection pressure. These variable regions may contribute to host adaptation, immune evasion, or vector interactions and merit further functional characterization. The time-calibrated phylogenetic reconstruction provided new insights into the evolutionary dynamics of *Ampelovirus* members, particularly GLRaV-1. The topology and divergence estimates indicate that GLRaV-1 is a recently diversified lineage, with shallow node heights and tight clustering suggesting rapid evolutionary radiation, likely driven by host adaptation, regional spread, or vector-mediated transmission. In contrast, GLRaV-3 exhibited deeper divergence, consistent with long-term co-evolution with grapevine or historical geographic dispersal. GLRaV-13 and LChV-2 displayed intermediate divergence, while GLRaV-4 formed a compact cluster indicative of low genetic variability, possibly reflecting a narrow host range, recent emergence, or limited geographic sampling.

The RelTime-derived divergence estimates, though relative, offer a temporal framework for evaluating *Ampelovirus* evolution in the absence of absolute calibration points. These patterns may reflect historical drivers such as clonal grapevine propagation, international plant trade, and the spread of mealybug and scale insect vectors. The high genetic variability within GLRaV-1 underscores the importance of genomic surveillance and molecular epidemiology, as distinct genotypes may differ in pathogenicity or transmission efficiency. Demographic analyses suggested historical population expansions, particularly in Clade I, potentially linked to grapevine domestication and anthropogenic dissemination. Although not statistically significant, these patterns align with known viticultural history. Overall, this study reveals strong genetic structuring, predominant purifying selection with localized diversifying pressure, and limited gene flow among GLRaV-1 populations in Iran. The findings highlight the need for continuous surveillance, region-specific management strategies, and further studies integrating broader sampling, vector identification, and host–virus interaction analyses to better anticipate the evolutionary potential of this economically important pathogen.
